# Supplementary material for: Sustained TNF signaling is required for the synaptic and anxiety-like behavioral response to acute stress
Source: Mol Psychiatry. 2022 Sep 14;27(11):4474–84. doi: 10.1038/s41380-022-01737-x (PMC9734040; doi:10.1038/s41380-022-01737-x)
Supplement: Supplementary file 1 — Supplemental Materials [file 41380_2022_1737_MOESM1_ESM.pdf]

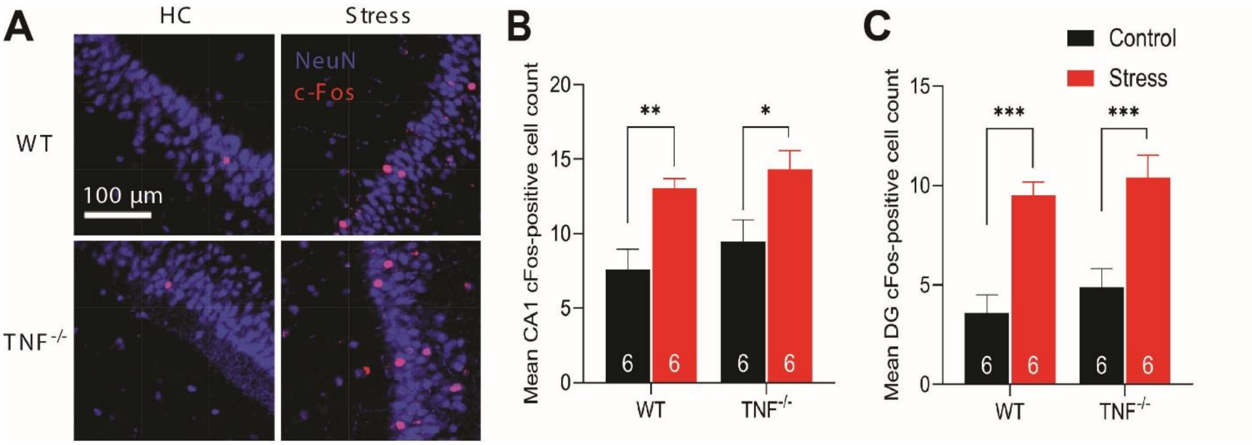

Supplemental Figure 1. (A) c-Fos labelling of the CA1 of the vHP in control and stressed animals. Labelling was significantly increased in the CA1 (**B**) and dentate gyrus (**C**) in both WT ($P < 0.001$) and TNF$^{-/-}$ ($P < 0.001$) animals following stress. There were no statistically significant differences between genotypes under control conditions ($P = 0.34$), nor following stress ($P = 0.51$).

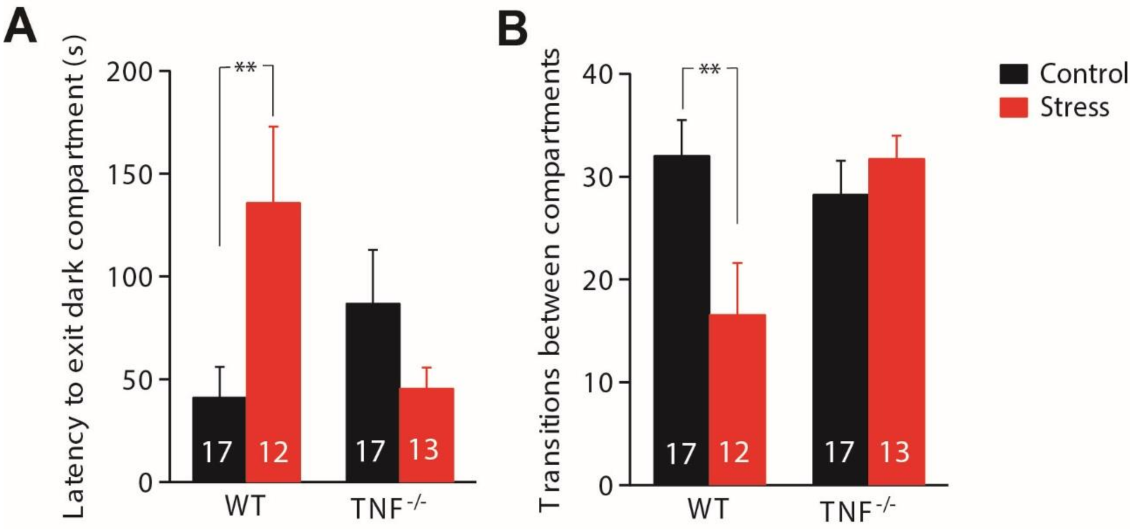

Supplemental Figure 2.

A. Latency to first exit in the light-dark box is longer in stressed wild-type (WT) animals compared to TNF knockout (TNF$^{-/-}$) mice (two-way ANOVA genotype x stress interaction $F (1, 49) = 9.140$, $P = 0.0040$; Tukey's *post hoc* analysis of control versus stress within WT $P = 0.0376$, TNF$^{-/-}$ $P = 0.4996$; Tukey's *post hoc* WT versus TNF$^{-/-}$ within controls $P = 0.3646$).

B. Stress reduces the number of transitions WT animals make from the dark box to the light box, while it does not affect the same parameter in TNF$^{-/-}$ mice (two-way ANOVA genotype x stress interaction $F (1, 55) = 6.992$, $P = 0.0107$; Tukey's *post hoc* analysis of control versus stress within WT $P = 0.0195$, TNF$^{-/-}$ $P = 0.8990$).

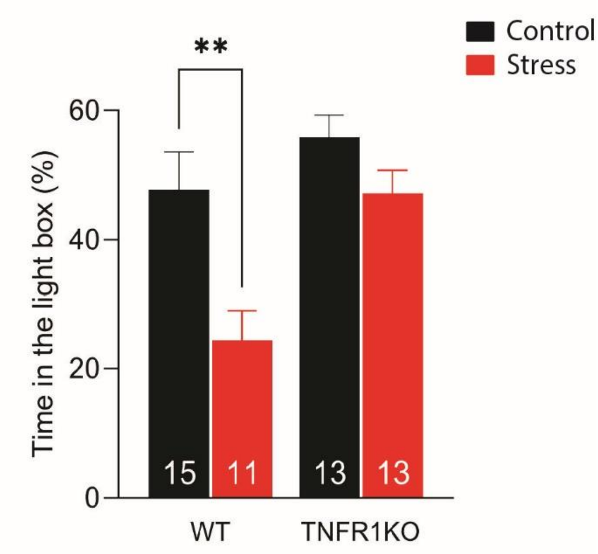

Supplemental Figure 3. No stress-induced anxiety-like behavior was observed in TNFR1KO animals (24 h post-stress; two-way ANOVA main effect of genotype F (1, 48) = 10.97, P = 0.0018, main effect of stress F (1, 48) = 11.73, P = 0.0013, genotype x stress interaction F (1, 48) = 2.434, P = 0.1253. Tukey’s post hoc analysis: difference within WT P = 0.0054, difference within TNFR1KO P = 0.5509).

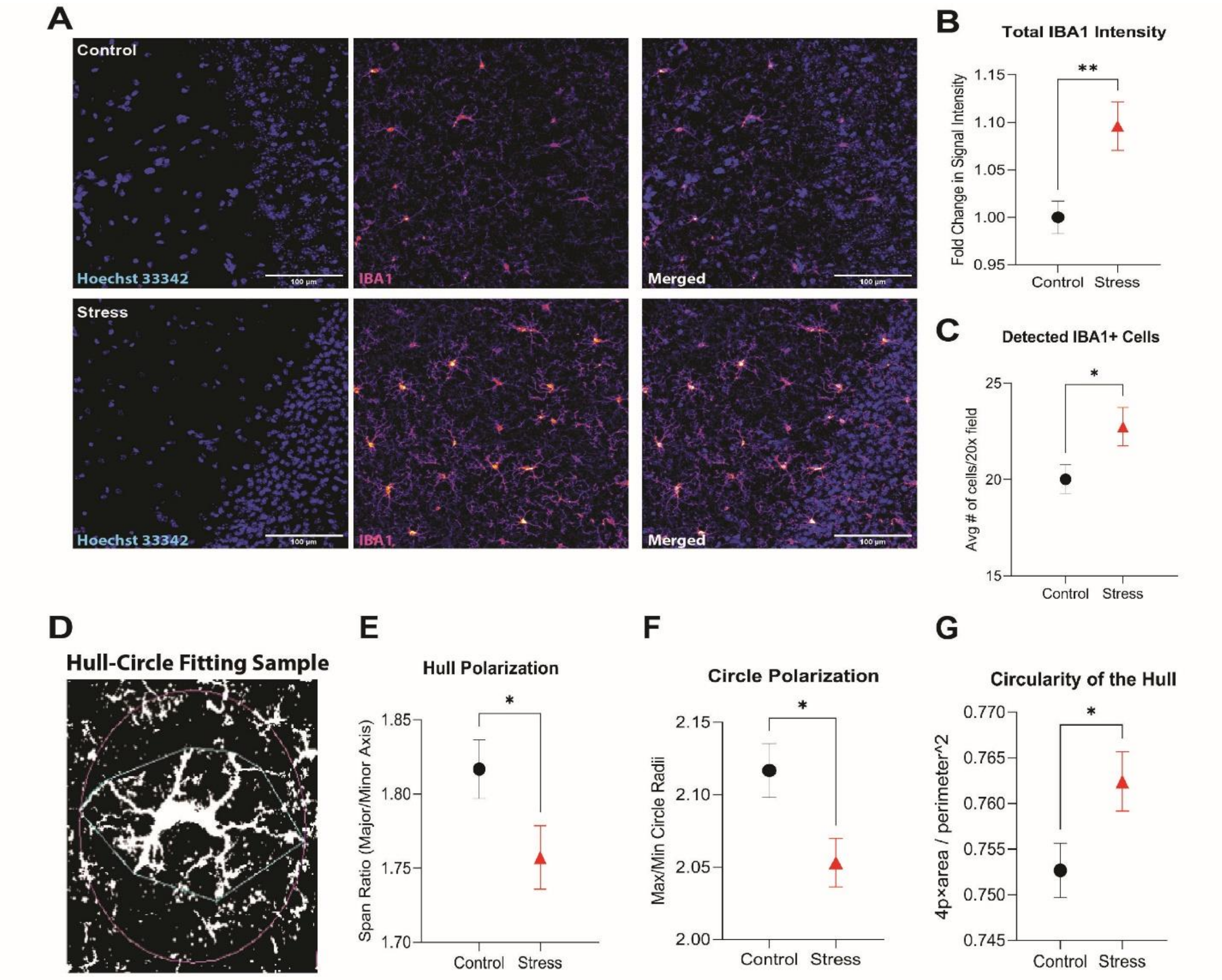

Supplemental Figure 4. A. Sample images demonstrating microglial activation as proxied by IBA1 signalling and cell density. **B.** Quantification of total IBA1 signa in the vHPC-CA1 region of unstressed controls and acutely stressed animals (4 h post-stress, student t-test, P = 0.0025, sample size n (number of animals N) for control = 43 (4), for stress = 45 (4)). **C.** Quantification of microglia in control versus stress (4 h post-stress, student t-test, P = 0.0327). **D.** An illustration of fitting microglia in a hull (inner green shape) and a circle (outer purple shape) for quantitative analysis of microglial morphology. The convex hull of a cell is the smallest convex set that encompass the cells. A convex set is a polygon where the line between any two points lies completely within the polygon. Microglia was delineated semi-automatically and the hull and circle shapes were fitted automatically using Frac-Lac package in ImageJ. **E-G.** Quantification of three parameters of the circularity of microglia, namely the polarization of the fitted hull (p = 0.0444), the polarization of the fitted circle (p = 0.0122), and the circularity of the hull (p = 0.03, all parameters were analyzed using a student t-test, n (N) for control = 43 (4), for stress = 45 (4), see the y axes for the equations used to measure each variable).

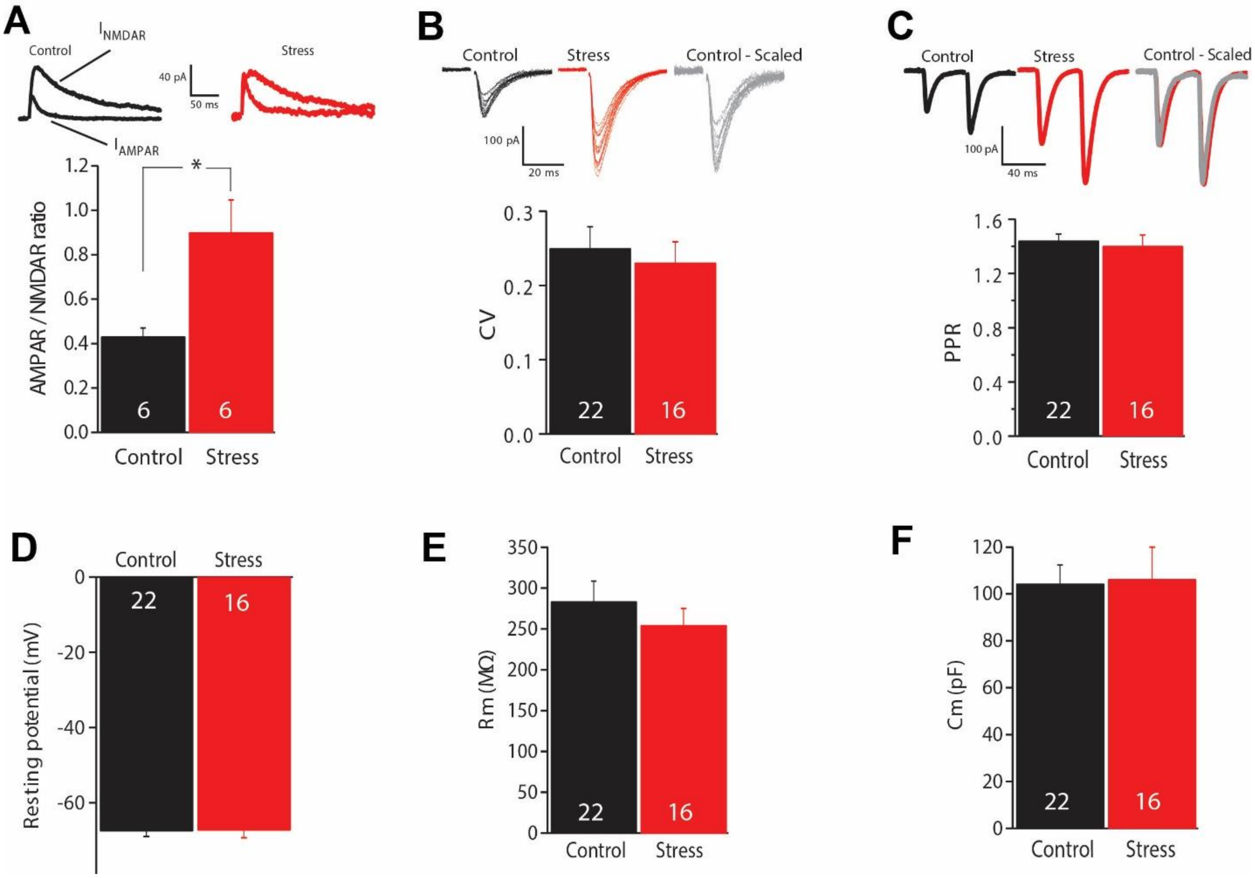

Supplemental Figure 5.
A. Cells in the baseline and 24 h post stress groups were perfused with D-AP5 subsequent to depolarization to +40 mV to isolate the AMPAR current. Example traces illustrate the isolated AMPAR current (black trace) which was subtracted from the compound current (not illustrated) to produce the resultant (isolated) NMDAR current (red trace). The peaks of the AMPAR current and NMDAR current were used in calculation of the AMPAR / NMDAR ratio data. Two-tailed t-test comparison of baseline (n = 6, N = 4) against the 24 h post stress is shown in figure S3.A. **B.** There were no significant changes in measures of pre-synaptic release probability, coefficient of variability (CV; P = 0.64) and **C.** paired pulse ratio (PPR; P = 0.66) (stress: n=16, N=8; control: n=22, N=15) measured 24 h post stress. Grey traces illustrate the example traces shown for control condition, scaled to match mean current amplitude for stress condition for comparison. The left panels illustrate individual synaptic response sweeps overlaid to highlight sweep-to-sweep variability. **D.** There were no significant differences between stress and control groups in resting membrane potential (P = 0.94), **E.** in membrane resistance (Rm; P = 0.40), nor in **F.** cell capacitance (Cm; P = 0.90). For all: stress n=16; N=8; control: n=22, N=15.

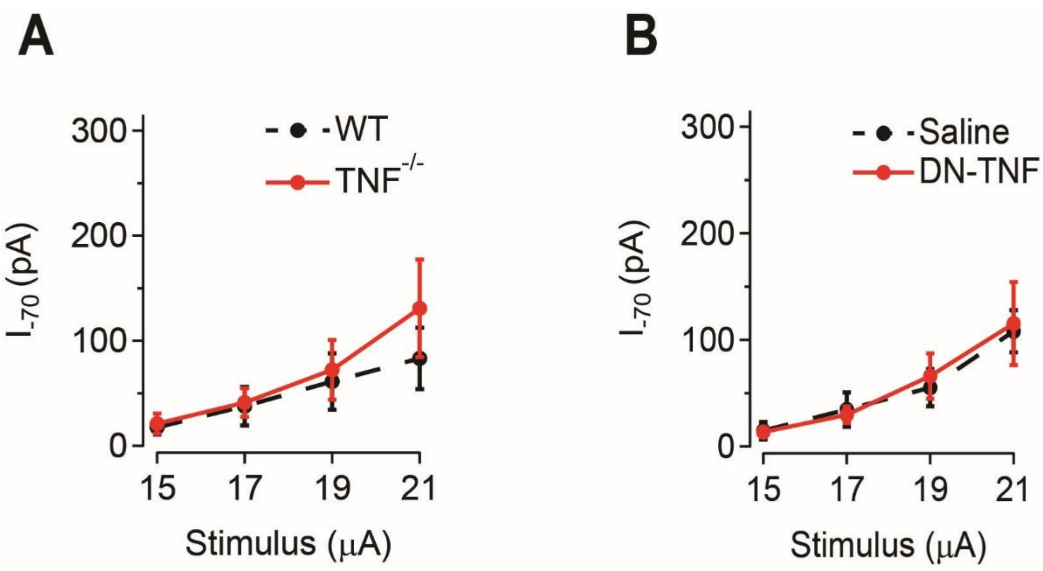

Supplemental Figure 6. A. There was no significant difference in basal AMPAR current in ventral hippocampus SC to CA1 synapses between WT animals and TNF$^{-/-}$ (P = 0.16; WT: n=7, N=5; *TNF$^{-/-}$:* n=6, N=3). **B.** Administration of DN-TNF did not alter basal AMPAR current relative to saline-injected controls (P = 0.58; Sal: n=18, N=12; DN-TNF: n=17, N=8).

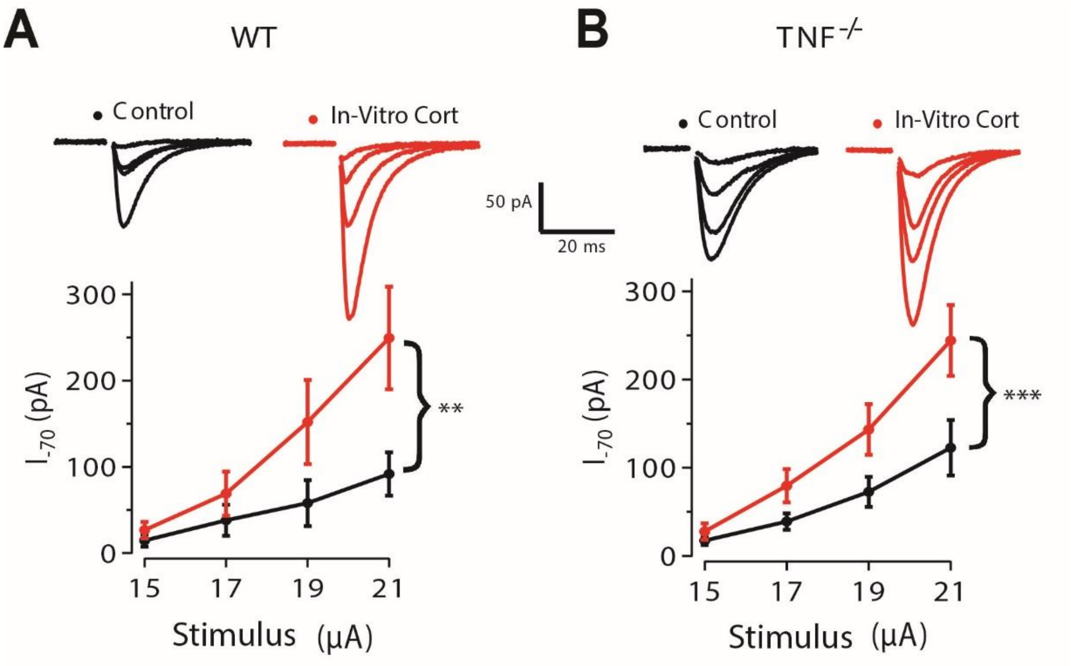

Supplemental Figure 7. A. Ex-vivo application of CORT induces synaptic potentiation of the vHPC-CA1 in both WT (Two-way ANOVA main effect of treatment F(1,34) = 8.3866, P = 0.0066; control: n=4, N = 4; CORT: n = 7, N = 4) and **B.** TNF$^{-/-}$ (Two-way ANOVA main effect of treatment F(1, 48) = 13.8841, P = 0.0005; control: n = 7, N = 5; CORT: n = 7, N = 5).

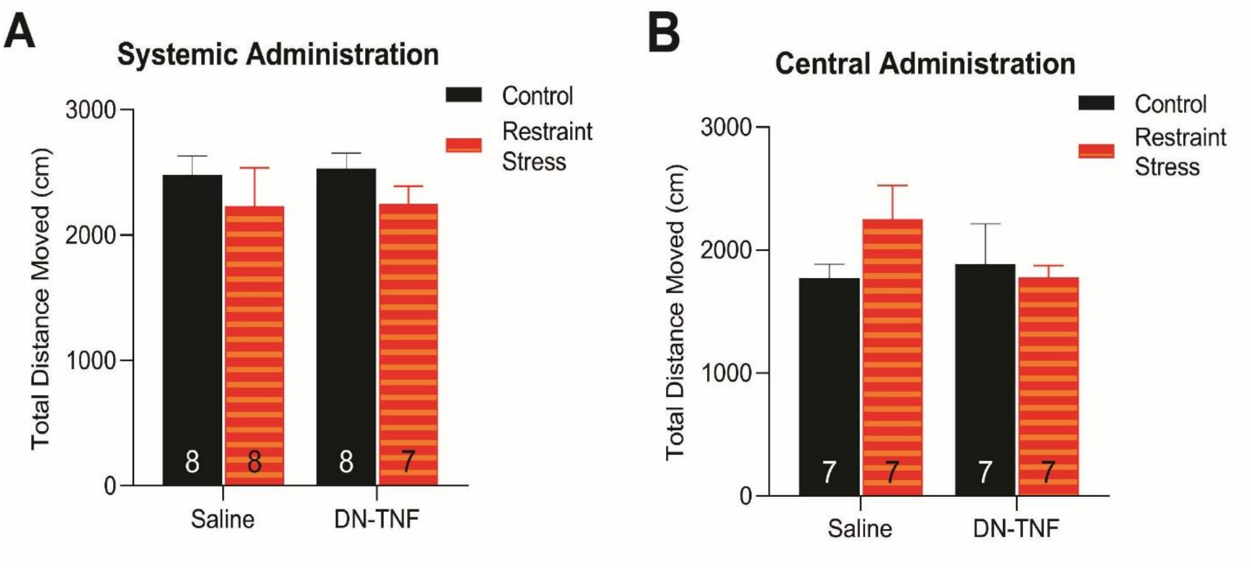

Supplemental Figure 8. A. The observed phenotype is not driven by the effects of the drug treatment on locomotion, as there was no difference in the distance travelled across the different groups (two-way ANOVA of drug treatment F (1, 27) = 0.02533, P = 0.8747; Tukey's post hoc analyses of control saline versus restraint stress (RS) saline P = 0.7978, control DN-TNF versus RS DN-TNF P = 0.7591). **B.** There is no difference in distance travelled under low anxiogenic conditions between the four groups, therefore, the observed behavioral effect of drug treatment is not mediated by differences in locomotive behavior (two-way ANOVA of the main effect of drug F (1, 24) = 0.6220, p =0.4380; Tukey's post hoc analyses of control saline versus RS saline P = 0.4359, control DN-TNF versus RS DN-TNF P = 0.9860).

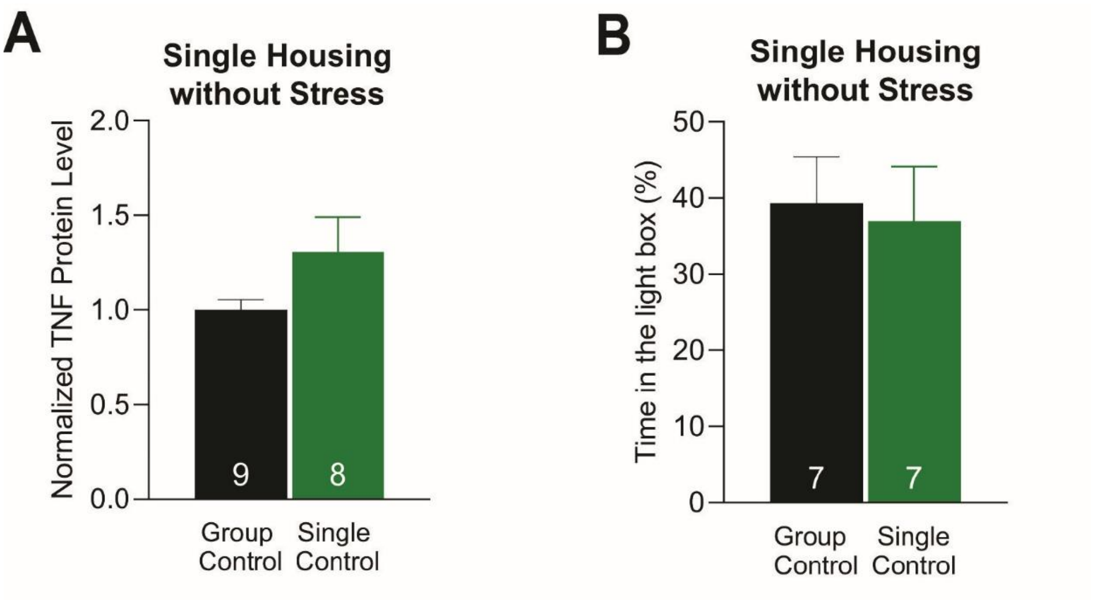

Supplemental Figure 9. A. There is no induction of vHPC levels of TNF in single-housed animals without stress (two-tailed student t-test, P = 0.1124). **B.** Single housing adult WT males over for 24 h does not induce anxiety-like behavior (two-tailed student t-test, P = 0.7999).

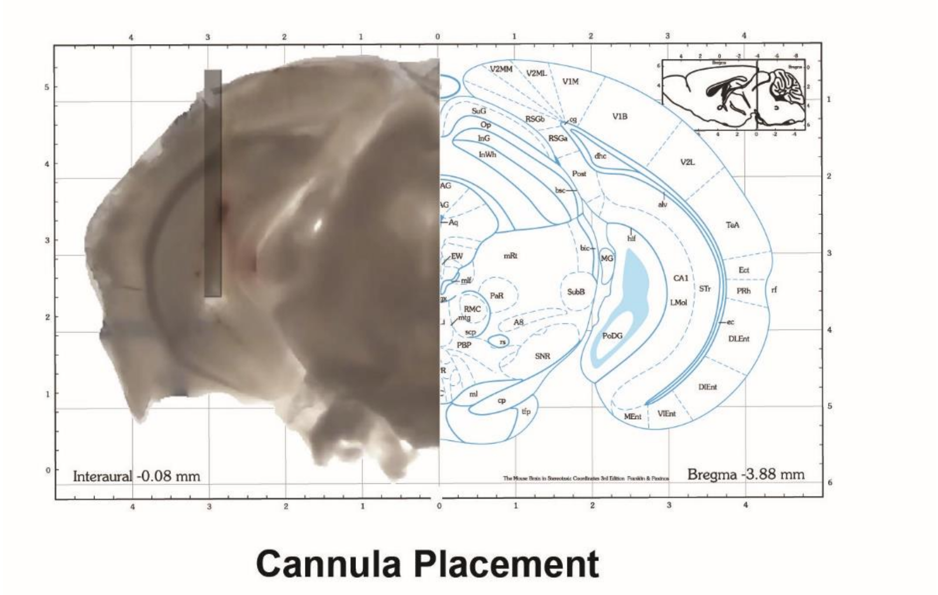

Supplemental Figure 10. A schematic and an image of the placement of the central cannula in the vHPC-CA1. The schematic portion of the figure was obtained from the Mouse Brain Atlas with permission.
